# Bekanntheit der Impfung gegen Humane Papillomviren (HPV) für Mädchen und Jungen in Deutschland – Ergebnisse der LIEBESLEBEN-Studie

**DOI:** 10.1007/s00103-026-04207-9

**Published:** 2026-03-05

**Authors:** Sarah Halbach, Catherin Bosle, Miriam Gerlich, Ursula von Rüden

**Affiliations:** 1 Referat Q3 – Evaluation, Methoden und Forschungsdaten, Bundesinstitut für Öffentliche Gesundheit (BIÖG), Maarweg 149–161, 50825 Köln, Deutschland; 2 Referat T3 – Sexuelle Gesundheit, Prävention von HIV und anderen STI, Bundesinstitut für Öffentliche Gesundheit (BIÖG), Köln, Deutschland

**Keywords:** HPV-Impfung, Sexuell übertragbare Infektionen, Impfbekanntheit, Impfkommunikation, Allgemeinbevölkerung, HPV vaccination, Sexually transmitted infections, Vaccination awareness, Vaccination communication, General population

## Abstract

**Hintergrund:**

Trotz geltender Impfempfehlung für Mädchen und Jungen gegen Infektionen mit Humanen Papillomviren (HPV) sind die Impfquoten in Deutschland niedrig. Die Bekanntheit der Impfung ist eine Grundvoraussetzung für das Impfverhalten und damit für eine Steigerung der Impfquoten. Die vorliegende Arbeit zielt darauf ab, die deutschlandweite Bekanntheit der HPV-Impfung jeweils für Mädchen und Jungen zu ermitteln sowie mögliche mit der Bekanntheit assoziierte Merkmale zur Soziodemografie, Familie und Beziehung und zum regionalen Kontext zu identifizieren. Die Ergebnisse dienen der Konzeption, Steuerung und Weiterentwicklung von Public-Health-Maßnahmen.

**Methoden:**

Im Rahmen der LIEBESLEBEN-Studie wurden bevölkerungsweite Daten mittels Online-Befragung erhoben und nach für Deutschland repräsentativen Kriterien gewichtet (*n* = 4640). Die Daten wurden mittels bivariater Analysen sowie multivariater logistischer Regressionsmodelle ausgewertet.

**Ergebnisse:**

Die HPV-Impfung für Mädchen ist 61,5 % der Befragten bekannt, die HPV-Impfung für Jungen kennen 32,6 %. Die Ergebnisse zeigen, dass die Impfbekanntheit, jeweils für Mädchen und Jungen, signifikant mit der Geschlechtsidentität, dem Bildungsstand, der sexuellen Orientierung und dem Zusammenleben mit Kindern assoziiert ist. Die Bekanntheit der Impfung für Mädchen ist zudem mit dem Beziehungsstatus und dem Grad der regionalen Deprivation assoziiert, die Bekanntheit der Impfung für Jungen mit dem Alter.

**Diskussion:**

In Deutschland existieren Bekanntheitsdefizite zur HPV-Impfung. Dies gilt insbesondere für die Impfung für Jungen. Kommunikationsmaßnahmen und Informationsangebote, die (lebensweltbezogene) Ansprache der Zielgruppen, die Einbindung von Fachakteurinnen und -akteuren und der Ausbau einer impfförderlichen Versorgungsstruktur sollten intensiviert werden.

## Abstract (en)

**Background:**

Despite the current recommendation of human papillomavirus (HPV) vaccination for girls and boys, vaccination rates in Germany remain low. Vaccination awareness is essential for vaccination behaviour and thus for an increase in vaccination rates. The aims of the present work were to investigate HPV vaccination awareness among the general population in Germany and to identify potential factors (sociodemographic, family and relationship characteristics, regional context) associated with HPV vaccination awareness. The results will help to conceptualize, manage, optimize and evaluate measures of public health.

**Methods:**

As part of the LIEBESLEBEN study, population-wide data were collected via an online survey (*n* = 4640). Data were weighted according to criteria representative for Germany. Bivariate analyses and multivariate logistic regression models were calculated.

**Results:**

In total, 61.5% of respondents are aware of HPV vaccination for girls and 32.6% are aware of HPV vaccination for boys. The results of regression analyses show that the awareness of HPV vaccination for girls and boys is significantly associated with gender, formal education level, sexual orientation and living with children. The awareness of HPV vaccination for girls is additionally associated with relationship status and the degree of regional deprivation. In addition, HPV vaccination awareness for boys is associated with age.

**Discussion:**

In Germany, HPV vaccination awareness is still modest. This is particularly true in terms of vaccination for boys. Measures to raise awareness should be intensified. These include communication and informational offerings, settings-based approaches to address target groups as well as involving (medical) professionals, and supporting a healthcare structure that promotes vaccination.

## Einleitung

Humane Papillomviren (HPV) zählen zu den häufigsten sexuell übertragbaren Erregern. Die Mehrheit der sexuell aktiven Menschen ist im Laufe ihres Lebens mindestens einmal von einer HPV-Infektion betroffen [1]. Es wird zwischen Low-Risk-HPV-Typen und High-Risk-HPV-Typen differenziert. Während Low-Risk-HPV-Typen u. a. Genitalwarzen verursachen, können als Folge von Infektionen mit High-Risk-HPV-Typen maligne Tumoren an Zervix, Vulva, Vagina, Penis, Oropharynx und Anus entstehen [1]. Der überwiegende Teil der HPV-Infektionen verläuft asymptomatisch und heilt selbstständig aus [1]. Bei etwa 10 % der Infektionen mit den High-Risk-Typen persistieren die Viren jedoch und können auch Jahre nach der Infektion zur Entstehung von Karzinomen führen [1]. In Deutschland erkranken jährlich etwa 7000 Frauen und 3000 Männer an HPV-assoziierten Karzinomen [2]. Nahezu alle Zervixkarzinome sind auf Infektionen mit HPV zurückzuführen [1].

Impfungen bieten einen effektiven Schutz vor einer Infektion mit den relevantesten HPV-Typen [3]. Dies gilt insbesondere dann, wenn die Impfung vor Beginn der sexuellen Aktivität erfolgt [4]. Die Ständige Impfkommission (STIKO) empfiehlt seit 2007 eine Impfung für Mädchen im Alter von 12 bis 17 Jahren bzw. seit 2014 im Alter von 9 bis 14 Jahren [4]. Erst seit 2018 werden auch Jungen einbezogen, sodass die HPV-Impfung seither als Standardimpfung für alle Kinder und Jugendlichen im Alter von 9 bis 14 Jahren in Deutschland empfohlen wird. Ungeimpfte Jugendliche sollen bis zum 18. Geburtstag nachgeimpft werden.

Bis 2021 stiegen die HPV-Impfquoten bei Jugendlichen in Deutschland kontinuierlich an. Seitdem stagnieren sie allerdings [5]. Im Jahr 2023 wiesen nur 55 % der 15-jährigen Mädchen und 34 % der 15-jährigen Jungen einen vollständigen Impfschutz auf [5]. Damit ist die HPV-Impfquote für 15-jährige Mädchen im europäischen Vergleich eher niedrig [6, 7] und liegt deutlich unterhalb der Ziele der Weltgesundheitsorganisation (WHO). In ihrer globalen Strategie zur Bekämpfung von Gebärmutterhalskrebs strebt die WHO eine Erhöhung der Impfquote auf mindestens 90 % bei Mädchen mit Vollendung des 15. Lebensjahres an [8]. Die Europäische Kommission setzt im Rahmen von „Europas Plan gegen den Krebs“ zusätzlich auf die Eindämmung anderer HPV-assoziierter Krebserkrankungen sowie auf eine deutliche Reduzierung der Virusübertragung und zielt in diesem Zusammenhang auch auf eine deutliche Steigerung der Impfquote bei Jungen [9].

Studien zeigen, dass die Sorge vor möglichen Nebenwirkungen des Impfstoffes, eine mangelnde öffentliche Aufklärung sowie ein geringes Wissen über die Impfung zu den zentralen Hindernissen gehören, sich selbst oder seine Kinder impfen zu lassen [10, 11]. Zur Steigerung der Impfquoten informiert das Bundesinstitut für Öffentliche Gesundheit (BIÖG) im Rahmen verschiedener Maßnahmen über HPV. So liefern z. B. die Portale *infektionsschutz.de* und *kindergesundheit-info.de* Informationen und Materialien zur HPV-Impfung [12, 13]. Auch die komplexe Intervention LIEBESLEBEN informiert bevölkerungsweit und zielgruppenspezifisch über HPV und unterstützt Fachkräfte durch Informationsmaterialien [14]. Außerdem war LIEBESLEBEN Teil der Initiative „PartnERship to Contrast HPV“ (PERCH, Laufzeit 11/2022–08/2025), die von der Europäischen Union (EU) gefördert wurde und an der sich 18 EU-Staaten beteiligten [15]. Die Initiative verfolgte u. a. das Ziel, das Wissen und Bewusstsein zu HPV zu fördern und die Impfquoten bei Mädchen und Jungen europaweit zu steigern.

Studien aus Deutschland haben sich mit der Bekanntheit der HPV-Impfung, insbesondere bei bestimmten Subgruppen, befasst. Für die Pilotstudie zur Studie „Gesundheit und Sexualität in Deutschland“ (GeSiD) wurden Frauen im Alter zwischen 18 und 35 Jahren dazu befragt, ob ihnen die HPV-Impfung für Mädchen bekannt sei [16]. Dies bejahte die überwiegende Mehrheit (80 %). Sharma et al. untersuchten die Bekanntheit der HPV-Impfung, wobei hier nicht zwischen der Impfung für Mädchen und derer für Jungen differenziert wurde [17]. Etwas weniger als die Hälfte (45 %) wusste von der Impfung. Frauen und Personen im Alter zwischen 25 und 34 Jahren war die Impfung häufiger bekannt. Das BIÖG befragt regelmäßig in einer bevölkerungsrepräsentativen Studie zum Infektionsschutz u. a. Eltern zur Bekanntheit von Impfungen für Kinder [18]. Die Ergebnisse aus dem Jahr 2020 zeigen, dass 79 % der Eltern die Impfempfehlung für Mädchen und 37 % die für Jungen kannten. In einer anderen Studie kannten 67 % der Eltern von Mädchen und 59 % der Eltern von Jungen die HPV-Impfung [19]. Eine weitere Untersuchung bei Jugendlichen zeigte, dass 65 % von der HPV-Impfung gehört hatten [20].

Auch systematische Übersichtsarbeiten zu europäischen Studien beschäftigten sich mit der Bekanntheit von HPV und der HPV-Impfung bei Jugendlichen [21] und Eltern [10]. Hierbei zeigen sich deutliche Länderunterschiede, die Autorinnen und Autoren schlussfolgern allerdings, dass insgesamt in Europa Handlungsbedarf zur Erhöhung der Bekanntheit von HPV und der HPV-Impfung besteht. Eine Studie aus den USA beobachtete einen Rückgang der Bekanntheit der HPV-Impfung bei Erwachsenen im Zeitraum zwischen 2013 bis 2018 [22]. Dort hatten im Jahr 2018 rund 60 % von der Impfung gegen HPV bzw. gegen Gebärmutterhalskrebs gehört, während es 2013 noch rund 67 % waren. Internationale Studien berichten zudem, dass die Bekanntheit von HPV und der HPV-Impfung u. a. nach Geschlecht, Alter und Bildung variiert [21, 22].

Die Bekanntheit der HPV-Impfung stellt eine Grundvoraussetzung für das Impfverhalten und damit für die Steigerung der Impfquoten dar. Vor diesem Hintergrund bedarf es regelmäßiger Untersuchungen zu ihrer Bekanntheit, um so die zielgruppenspezifische Konzeption, Steuerung, Weiterentwicklung und Bewertung von Public-Health-Maßnahmen zu unterstützen. Hierfür ist das differenzierte Verständnis der Bekanntheit sowohl in der Gesamtbevölkerung als auch in den Zielgruppen erforderlich.

Anknüpfend an die Zielsetzungen der Studie „AIDS im Öffentlichen Bewusstsein der Bundesrepublik Deutschland“ und in Ergänzung um weitere Aspekte des WHO-Ansatzes zur sexuellen Gesundheit [23] führte die damalige Bundeszentrale für gesundheitliche Aufklärung (BZgA, jetzt BIÖG) im Jahr 2023 erstmals die *LIEBESLEBEN-Studie *[24] durch. Ziel der Studie war es, das Wissen, die Einstellungen und das Verhalten zu sexueller Gesundheit und sexuell übertragbaren Infektionen (STI) in der deutschsprachigen Allgemeinbevölkerung zu ermitteln. Neben der Kenntnis der Impfung für Mädchen wurden hier auch bevölkerungsweite Daten zur Kenntnis der Impfung für Jungen erhoben, um mögliche Unterschiede in der Bekanntheit der Impfangebote zu eruieren und differenziert zu adressieren. Nachfolgend werden diese dezidiert berichtet. Es werden folgende Fragestellungen beantwortet:

- Wie hoch ist die Bekanntheit der HPV-Impfung für Mädchen?
- Wie hoch ist die Bekanntheit der HPV-Impfung für Jungen?
- Inwieweit ist die Bekanntheit der HPV-Impfung für Mädchen mit Merkmalen zur Soziodemografie, Familie und Beziehung sowie zum regionalen Kontext assoziiert?
- Inwieweit ist die Bekanntheit der HPV-Impfung für Jungen mit Merkmalen zur Soziodemografie, Familie und Beziehung sowie zum regionalen Kontext assoziiert?

## Methoden

### Studie, Stichprobe und Datenerhebung

Die LIEBESLEBEN-Studie ist eine bevölkerungsweite CAWI-Befragung (Computer Assisted Web Interview). Ihre Grundgesamtheit umfasst alle in einem aktiv rekrutierten Online-Access-Panel angemeldeten Personen (deutschsprachig) ab 16 Jahren. Die Stichprobenziehung erfolgte quotiert nach den Merkmalen Bildung, Alter, Geschlecht und Wohnort. Da Personen im Alter zwischen 16 und 44 Jahren als Risikogruppe für STI gelten, wurde diese Altersspanne disproportional überquotiert. Die Basisstichprobe bestand aus *n* = 4040 Personen. Sie wurde um *n* = 600 Personen mit den sexuellen Orientierungen bisexuell, pansexuell, vorwiegend/ausschließlich homosexuell, asexuell oder uneindeutig/unsicher aufgestockt, um auch für diese Personengruppen zuverlässige Ergebnisse zu generieren. Daraus ergab sich eine Gesamtstichprobe von *n* = 4640 Personen. Die Datenerhebungen erfolgten durch INFO GmbH Markt- und Meinungsforschung.

Der Fragebogen umfasste Fragen zu Wissen, Einstellungen und Verhalten in Bezug auf STI und weitere Aspekte der sexuellen Gesundheit. Im Anschluss an einen Fragebogenpretest erfolgte die Datenerhebung, die durchschnittlich 21 min dauerte. Die Teilnehmenden erhielten eine Incentivierung. Maßnahmen zur Qualitätssicherung während der Panelrekrutierung und Befragungsdurchführung sowie weitere Informationen zur Studie sind an anderen Stellen beschrieben [24, 25].

### Untersuchte Variablen

#### Bekanntheit der HPV-Impfung für Mädchen.

Diese Variable wurde wie folgt erfasst: „War Ihnen vor diesem Interview bekannt, dass sich Mädchen/junge Frauen zwischen 9 und 17 Jahren kostenlos gegen humane Papillomviren (HPV), z. B. zum Schutz vor Gebärmutterhalskrebs, impfen lassen können?“ Die Antwortkategorien wurden codiert mit 0 = „nein“, 1 = „ja“ und 2 = „keine Angabe“. Die Kategorie „keine Angabe“ wurde aufgrund ihrer geringen Fallzahl aus den Analysen ausgeschlossen.

#### Bekanntheit der HPV-Impfung für Jungen.

Diese Variable wurde wie folgt erhoben: „War Ihnen vor diesem Interview bekannt, dass sich Jungen/junge Männer zwischen 9 und 17 Jahren kostenlos gegen humane Papillomviren (HPV) zum Schutz vor HPV-bedingten Krebsarten impfen lassen können?“ Die Antwortkategorien wurden codiert mit 0 = „nein“, 1 = „ja“ und 2 = „keine Angabe“, wobei die Kategorie „keine Angabe“ aufgrund ihrer geringen Fallzahl aus den Analysen ausgeschlossen wurde.

#### Merkmale zur Soziodemografie.

Folgende Variablen wurden einbezogen: Alter („16–25 Jahre“, „26–35 Jahre“, „36–45 Jahre“, „46–55 Jahre“, „56–65 Jahre“, „66–75 Jahre“ und „76+ Jahre“), formaler Bildungsstand („kein Schulabschluss/(angestrebter) Hauptschulabschluss“, „(angestrebter) Realschulabschluss“, „(angestrebtes) (Fach‑)Abitur/(Fach‑)Hochschulabschluss“) und Geschlecht. Dieses wurde auf Basis der Selbstidentifikation erhoben. Aufgrund der zu geringen Datenbasis von Personen, die sich als „nicht-binär/genderqueer“, „inter*/divers“ oder „agender“ identifizieren bzw. eine andere offene Angabe oder „keine Angabe“ machten (*n* = 41), konnten in die statistischen Modelle lediglich die Geschlechtsidentifikationen „weiblich“ und „männlich“ aufgenommen werden.

#### Merkmale zur Beziehung und Familie.

Hier flossen die folgenden Variablen ein: sexuelle Orientierung („heterosexuell“ und „LSBA = lesbisch, schwul, bisexuell, asexuell und weitere“), Beziehung („keine feste Beziehung“, „feste Beziehung“) und Kinder unter 18 Jahre im Haushalt („nein“, „ja“, „keine Angabe“).

#### Merkmale zum regionalen Kontext.

Folgende Variablen wurden aufgenommen: Wohnortgröße („Kleinstadt/Landgemeinde“, „Mittelstadt“, „Großstadt“), Region („Ost“, „West“) und Deprivationsindex („geringe Deprivation = Quintil 1“, „mittlere Deprivation = Quintil 2–4“, „hohe Deprivation = Quintil 5“, „keine Angabe“). Der Deprivationsindex (German Index of Socioeconomic Deprivation GISD; [26, 27]) bildet auf der Grundlage von Daten zu Bildung, Beruf und Einkommen regionale sozioökonomische Unterschiede ab und erlaubt darüber die Darstellung sozialer Benachteiligung. Er wurde über die regionale Kennung (Gemeindeebene) hinzugefügt. Die Kategorie „keine Angabe“ wurde aufgrund ihrer geringen Fallzahl aus den Analysen ausgeschlossen.

### Statistische Analysen

Für die vorliegenden Analysen wurden die Daten nach repräsentativen Kriterien für die Allgemeinbevölkerung Deutschlands gewichtet (Merkmale: Alter, Geschlecht, Bildung, Haushaltsgröße und Bundesland), um die Stichprobenverteilung an die Sollstrukturen entsprechend dem Mikrozensus des Statistischen Bundesamtes (2022) anzupassen [28]. Zudem wurden die Disproportionalität in der Altersverteilung und die Aufstockung um verschiedene sexuelle Orientierungen durch Gewichtungsverfahren an die realen Anteile in der Grundgesamtheit angepasst.

Die Daten wurden mithilfe des Moduls „Complex Samples“ der Datenanalysesoftware IBM SPSS Statistics 30.0.0.0. (IBM, Armonk, NY, USA) auf Basis der gewichteten und stratifizierten Stichprobe analysiert. Nach Ausschluss der oben beschriebenen Fälle umfasst die Stichprobe *n* = 4472 Personen. Prozentangaben dienen der Beschreibung der uni- und bivariaten Prävalenzen für kategoriale Variablen. Mithilfe bivariater Analysen (Chi-Quadrat-Tests; Signifikanzniveau *p* ≤ 0,05) wurden signifikante Gruppenunterschiede ermittelt. Um Zusammenhänge der unabhängigen Variablen mit den jeweils abhängigen Variablen zu untersuchen, wurden multivariate logistische Regressionsmodelle berechnet. Als abhängige Variablen wurden (1) die Bekanntheit der HPV-Impfung für Mädchen und (2) die Bekanntheit der HPV-Impfung für Jungen einbezogen. Die Ergebnisse werden in Form von Odds Ratios (OR) mit zugehörigen Konfidenzintervallen (KI) berichtet.

## Ergebnisse

Die Merkmale der Stichprobe sind in Tab. 1 dargestellt. Insgesamt kennen 61,5 % der bevölkerungsweit Befragten im Jahr 2023 die HPV-Impfung für Mädchen und 32,6 % die Impfung für Jungen.

**Tab. 1:** Deskription der Stichprobe (*n* = 4640)

		Absolute Häufigkeit (ungewichtet)	Absolute Häufigkeit (gewichtet)	Relative Häufigkeit (gewichtet %)
Bekanntheit der Impfung gegen Humane Papillomviren (HPV) für Mädchen	Nein	1616	1725	37,2
	Ja	2961	2853	61,5
	Keine Angabe^a^	63	62	1,3
Bekanntheit HPV-Impfung für Jungen	Nein	2953	3069	66,1
	Ja	1633	1513	32,6
	Keine Angabe^a^	54	58	1,2
Alter	16–25 Jahre	857	558	12,0
	26–35 Jahre	1104	699	15,1
	36–45 Jahre	1051	673	14,5
	46–55 Jahre	476	733	15,8
	56–65 Jahre	529	867	18,7
	66–75 Jahre	378	640	13,8
	76 Jahre und älter	245	469	10,1
Geschlechtsidentität	Weiblich	2372	2358	50,8
	Männlich	2227	2263	48,8
	Nichtbinär/genderqueer, inter*/divers, agender, weitere, keine Angabe^a^	41	19	0,4
Formaler Bildungsstand	Kein Schulabschluss/(angestrebter) Hauptschulabschluss	586	652	14,0
	(Angestrebter) Realschulabschluss	2106	2245	48,4
	(Angestrebtes) (Fach‑)Abitur/(Fach-)Hochschulabschluss	1948	1743	37,6
Sexuelle Orientierung	Heterosexuell	3669	4128	89,0
	LSBA (lesbisch, schwul, bisexuell, asexuell) und weitere	785	292	6,3
	Keine Angabe	186	220	4,7
Beziehungsstatus	Keine feste Beziehung	1544	1417	30,5
	Feste Beziehung	3096	3223	69,5
Kinder unter 18 im Haushalt	Nein	3451	3487	75,1
	Ja	1099	1048	22,6
	Keine Angabe	90	106	2,3
Wohnortgröße	Kleinstadt/Landgemeinde ^b^	1647	1758	37,9
	Mittelstadt ^c^	1227	1226	26,4
	Großstadt ^d^	1766	1657	35,7
Region	Alte Bundesländer, einschl. Westberlin	3810	3850	83,0
	Neue Bundesländer, einschl. Ostberlin	830	790	17,0
Deprivationsindex	Gering (1)	1493	1520	32,8
	Mittel (2–4)	2475	2464	53,1
	Hoch (5)	630	618	13,3
	Keine Angabe^a^	42	38	0,8

^a^ Diese Kategorien werden aufgrund einer zu geringen Fallzahl aus allen bi- und multivariaten Analysen ausgeschlossen

^b^ bis unter 20.000 Einwohnerinnen und Einwohner

^c^ 20.000 bis unter 100.000 Einwohnerinnen und Einwohner

^d^ 100.000 Einwohnerinnen und Einwohner und mehr

### Ergebnisse der bivariaten Analysen

**Abb. 1:**
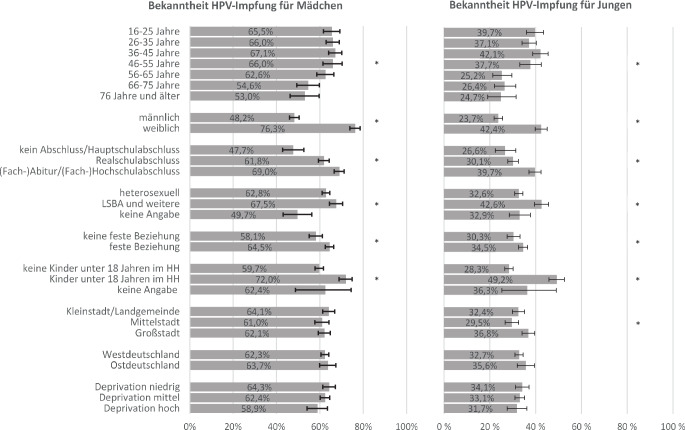
Verteilung der Bekanntheit der HPV-Impfung jeweils für Mädchen und Jungen nach Indikatoren zu Soziodemografie, Familie und Beziehung sowie regionalem Kontext (in % (inkl. 95 % Konfidenzintervall), gewichtete Stichprobe, *n* = 4472). *LSBA* lesbisch, schwul, bisexuell, asexuell; *HH* Haushalt; * signifikante Gruppenunterschiede (*p* ≤ 0,05)

Abb. 1 zeigt die Gruppenunterschiede zu den Bekanntheitswerten der Impfungen jeweils für Mädchen und für Jungen.

#### Bekanntheit der Impfung für Mädchen

Die Bekanntheit der Impfung für Mädchen unterscheidet sich signifikant zwischen den Altersgruppen (*p* ≤ 0,05). In den Altersgruppen unter 56 Jahren zeigen sich mit 65,5–67,1 % relativ ähnliche Bekanntheitswerte, während die Impfung mit 53,0–54,6 % in den Gruppen ab 66 Jahren signifikant unbekannter ist. Insgesamt am bekanntesten ist die HPV-Impfung für Mädchen bei Frauen. Während 76,3 % der Frauen sie kennen, ist der Anteil bei den Männern mit 48,2 % signifikant geringer (*p* ≤ 0,05). Es zeigen sich außerdem signifikante Unterschiede nach formalem Bildungsstand (*p* ≤ 0,05): So ist die Impfung mit lediglich 47,7 % am unbekanntesten bei Personen ohne Schulabschluss bzw. mit (angestrebtem) Hauptschulabschluss, während 69,0 % der Personen mit (angestrebtem) (Fach‑)Abitur bzw. (Fach‑)Hochschulabschluss von der Impfung gehört haben. Rund 67,5 % der Personen mit den sexuellen Orientierungen „LSBA und weitere“ kennen die HPV-Impfung für Mädchen. Das ist ein signifikant höherer Anteil als bei heterosexuellen Personen mit 62,8 % (*p* ≤ 0,05). Außerdem geht der Beziehungsstatus „feste Beziehung“ mit einer signifikant höheren Bekanntheit einher (64,5 % vs. 58,1 %; *p* ≤ 0,05). Ein signifikanter Unterschied liegt zudem für das Zusammenleben mit Kindern unter 18 Jahren vor (*p* ≤ 0,05): So kennen 72,0 % dieser Personen die Impfung, während sie bei Personen in kinderlosen Haushalten lediglich bei einem Anteil von 59,7 % bekannt ist.

#### Bekanntheit der Impfung für Jungen

Der Anteil derer, die die HPV-Impfung für Jungen kennen, ist in der Altersgruppe der 36- bis 45-Jährigen mit 42,1 % am höchsten, gefolgt von der Altersgruppe der 16- bis 25-Jährigen mit 39,7 %. Die Bekanntheit nimmt ab einem Alter von 56 Jahren deutlich ab (*p* ≤ 0,05). Die Impfung für Jungen ist bei 42,4 % der Frauen bekannt, während Männer sie signifikant weniger kennen (23,7 %, *p* ≤ 0,05). Außerdem ergeben sich signifikante Unterschiede nach formalem Bildungsstand (*p* ≤ 0,05): Personen mit (angestrebtem) (Fach‑)Abitur bzw. (Fach‑)Hochschulabschluss kennen die Impfung signifikant häufiger als Personen ohne Schulabschluss bzw. mit (angestrebtem) Hauptschulabschluss (39,7 % vs. 26,6 %). Insgesamt 42,6 % der Personen mit einer der sexuellen Orientierungen „LSBA und weitere“ kennen die Impfung für Jungen, unter heterosexuellen Personen sind es mit 32,6 % signifikant weniger (*p* ≤ 0,05). Außerdem geht der Beziehungsstatus „feste Beziehung“ mit einer signifikant höheren Bekanntheit einher (34,5 % vs. 30,3 %; *p* ≤ 0,05). Unter Personen mit Kindern unter 18 Jahren im Haushalt ist die Impfung mit 49,2 % insgesamt am bekanntesten (vs. 28,3 % in kinderlosen Haushalten, *p* ≤ 0,05). Personen, die in Großstädten leben, kennen die Impfung signifikant häufiger als Personen, die in Mittelstädten leben (36,8 % vs. 29,5 %; *p* ≤ 0,05).

### Ergebnisse der multivariaten Analysen

**Abb. 2:**
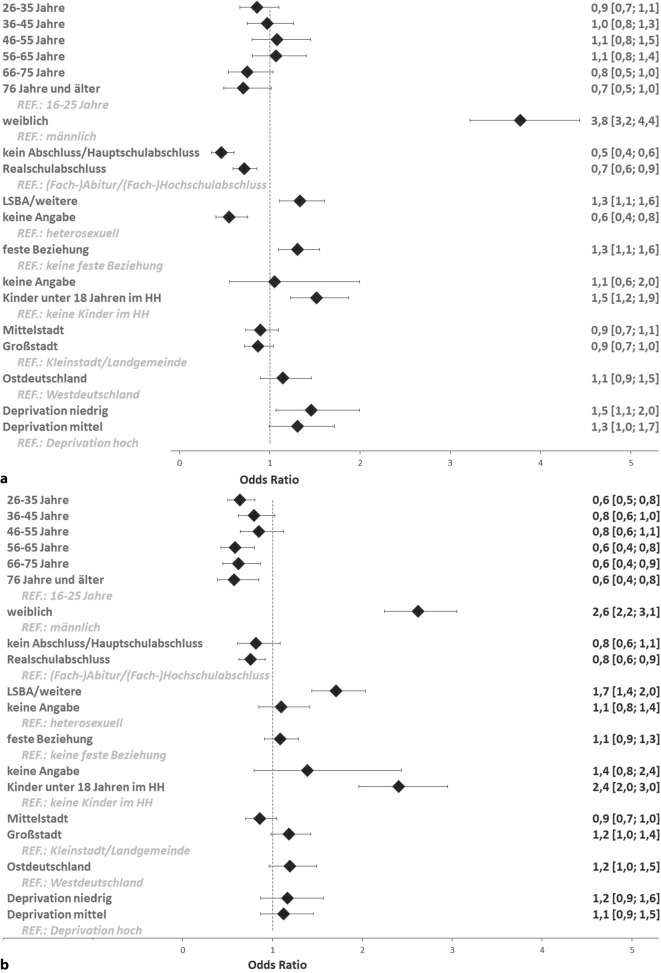
Multivariate logistische Regressionsmodelle zum Zusammenhang der Indikatoren zu Soziodemografie, Familie und Beziehung sowie regionalem Kontext mit der Bekanntheit der HPV-Impfung (**a**) für Mädchen und (**b**) für Jungen (gewichtete Stichprobe, *n* = 4472). Berichtet werden Odds Ratios sowie je obere und untere Konfidenzintervalle. *HH* Haushalt; *LSBA* lesbisch, schwul, bisexuell, asexuell; *REF* Referenzgruppe

In Abb. 2a sind die Ergebnisse der Regressionsanalyse zur Bekanntheit der HPV-Impfung für Mädchen dargestellt. Abb. 2b zeigt die Ergebnisse zur Bekanntheit der Impfung für Jungen.

#### Bekanntheit der Impfung für Mädchen

Es liegen signifikante Zusammenhänge mit der Geschlechtsidentität und dem Bildungsstand vor. So ist die Chance, dass Frauen die Impfung kennen (OR = 3,8), deutlich höher als die von Männern. Personen ohne Schul- oder mit (angestrebtem) Hauptschulabschluss (OR = 0,5) und Personen mit (angestrebtem) Realschulabschluss (OR = 0,7) haben signifikant geringere Chancen, die Impfung für Mädchen zu kennen, als Personen mit (angestrebtem) (Fach‑)Abitur oder (Fach‑)Hochschulabschluss. Personen mit den sexuellen Orientierungen „LSBA und weitere“ (OR = 1,3) wissen eher von der Impfung als heterosexuelle Personen. Auch Personen in fester Beziehung (OR = 1,3) und Personen, die mit Kindern unter 18 Jahren in einem Haushalt leben (OR = 1,5), kennen die Impfung eher als ihre jeweiligen Referenzgruppen. Zudem kennen Personen, die in Regionen mit niedriger Deprivation leben (OR = 1,5), die Impfung eher als Personen aus Regionen mit hoher Deprivation.

#### Bekanntheit der Impfung für Jungen

Befragte in der jüngsten Altersgruppe der 16- bis 25-Jährigen kennen die Impfung eher als Personen in den Altersgruppen 26–35 Jahre (OR = 0,6), 56–65 Jahre (OR = 0,6), 66–75 Jahre (OR = 0,6) und 76 Jahre und älter (OR = 0,6). Frauen haben eine höhere Chance, die Impfung für Jungen zu kennen (OR = 2,6), als Männer. Personen mit (angestrebtem) Realschulabschluss haben eine geringere Chance, die Impfung für Jungen zu kennen (OR = 0,8), verglichen mit den höheren Bildungsabschlüssen. Für die Indikatoren zur Familie und Beziehung liegen signifikante Zusammenhänge mit der sexuellen Orientierung und dem Zusammenleben mit Kindern unter 18 Jahren vor. Menschen mit den sexuellen Orientierungen „LSBA und weitere“ haben eine höhere Chance, die Impfung zu kennen (OR = 1,7), als heterosexuelle Personen. Personen, die in Haushalten mit Kindern unter 18 Jahren leben, kennen die Impfung eher (OR = 2,4) als Personen in kinderlosen Haushalten.

## Diskussion

### Bekanntheit der HPV-Impfung für Mädchen und Jungen

Die Bekanntheit der HPV-Impfung ist eine Grundvoraussetzung zur Steigerung der Impfquoten. Die vorliegenden Ergebnisse zeigen jedoch, dass lediglich 61,5 % der deutschlandweit Befragten die Impfung für Mädchen kennen. Die Bekanntheit der Impfung für Jungen liegt sogar deutlich darunter: Lediglich ein Drittel kennt diese (32,6 %).

Ähnliche Bekanntheitswerte der Impfung für Mädchen zeigen die Ergebnisse einer bevölkerungsweiten Studie aus den USA [22]. Dort gaben 60 % der Erwachsenen an, die Impfung gegen HPV bzw. gegen Gebärmutterhalskrebs zu kennen. In einer deutschlandweiten Befragung wussten nur rund 45 % der Menschen von der Impfung gegen HPV [17]. Konträr zu unserer Erhebung wurde dort nicht zwischen der Impfung für Mädchen und Jungen differenziert, was die direkte Vergleichbarkeit mit unseren Ergebnissen einschränkt.

Die vorliegende Diskrepanz in den Bekanntheitswerten der Impfungen für Mädchen und Jungen deutet darauf hin, dass die HPV-Impfung in der öffentlichen Wahrnehmung auch 5 Jahre nach der geschlechtsneutralen Impfempfehlung noch immer primär mit dem weiblichen Geschlecht bzw. Gebärmutterhalskrebs assoziiert ist. Eine Studie, die das Wissen zu HPV unter deutschen Studierenden untersuchte, konnte zeigen, dass es etwa 22 % der Befragten nicht bekannt war, dass die HPV-Impfung auch Jungen schützt [29].

Darüber hinaus zeigen unsere Ergebnisse eine besonders geringe Bekanntheit bei Männern. Dies betrifft sowohl die Impfung für Mädchen als auch die für Jungen. Auch unter Konstanthaltung aller anderen Indikatoren bleibt dieses Ergebnis bestehen. Männer gehören daher zu einer Risikogruppe dafür, die Impfungen nicht zu kennen. Ähnliches gilt für Menschen mit einem niedrigeren formalen Bildungsstand. Solche geschlechts- und bildungsabhängigen Unterschiede korrespondieren mit den Ergebnissen anderer Studien [21, 22]. Die vorliegende Untersuchung zeigt außerdem altersassoziierte Zusammenhänge für die Bekanntheit der Impfung für Jungen, während die Bekanntheit der Impfung für Mädchen altersunabhängig ist. Hier ist positiv anzumerken, dass die jüngste Altersgruppe (16–25 Jahre), die größtenteils bereits zu den Adressaten für die geschlechtsneutrale Impfempfehlung ab 2018 gehört, insgesamt besser über die Impfung für Jungen informiert zu sein scheint. Dieses Ergebnis ist unabhängig von der eigenen Geschlechtsidentität. Es ist daher zu vermuten, dass diese Zielgruppe von bestehenden Kommunikationsmaßnahmen sowie Informationen zur geschlechtsneutralen Impfstrategie im Rahmen der medizinischen Versorgung in Teilen erreicht wurde.

Weiterhin positiv zu verzeichnen ist, dass die Impfung für Mädchen und Jungen bei Personen, die mit Kindern unter 18 Jahren zusammenleben, eher bekannt ist. In dieser Gruppe kennen 72,0 % die Impfung für Mädchen und 49,2 % die für Jungen. Es ist anzunehmen, dass sich in der benannten Gruppe zum großen Teil Eltern bzw. sorgeberechtigte Personen von Kindern im impffähigen Alter befinden, die damit zu einer zentralen Zielgruppe von Kommunikationsmaßnahmen zu HPV gehören. Die Studie zum Infektionsschutz erfasst die Bekanntheit der HPV-Impfempfehlung jeweils für Mädchen und Jungen bei Eltern von Kindern im Alter von 0 bis 13 Jahren [18]. Es wurde gezeigt, dass im Jahr 2020 79 % der befragten Eltern die Impfempfehlung für Mädchen und 37 % die Impfempfehlung für Jungen kannten. In einer Online-Befragung von Eltern 9‑ bis 14-jähriger Kinder lagen die Bekanntheitswerte der HPV-Impfung bei Eltern von Mädchen bei 67 % und bei Eltern von Jungen bei 59 % [19].

Weiterhin zeigen unsere Ergebnisse, dass Personen mit den sexuellen Orientierungen „LSBA und weitere“ die Impfung für Mädchen und Jungen eher kennen. Ein ähnliches Ergebnis zeigte die GeSiD-Studie. Personen mit den sexuellen Orientierungen „LSBA“ kannten mit höherer Chance mehr STI als heterosexuelle Personen [30]. Darüber hinaus ist die HPV-Impfung für Mädchen bei Personen in festen Beziehungen eher bekannt. Zu einem ähnlichen Ergebnis kam eine Studie aus Italien [31]. Dort hatten Personen, die in einer festen Beziehung lebten, eher von HPV gehört.

Außerdem ist die Impfung für Mädchen bei Personen aus Regionen mit niedriger Deprivation eher bekannt als bei Personen aus Regionen mit hoher Deprivation. Dieser Zusammenhang zeigt sich nicht für die Bekanntheit der Impfung für Jungen. Dies sollte im Rahmen weiterer Studien untersucht werden.

### Praktische Implikationen

Um die Bekanntheit der HPV-Impfung zu erhöhen, sind weitere Maßnahmen zur Impfkommunikation erforderlich und bereits bestehende Maßnahmen sind zu intensivieren. Angebote zur Impfkommunikation für die Allgemeinbevölkerung und zielgruppenspezifische Informationsangebote durch staatliche und nichtstaatliche Institutionen gelten als wichtiger Bestandteil einer Gesamtstrategie zur Förderung des Impfwissens [33]. Hierzu gehören u. a. Aufklärungsaktionen, Informationen auf den Internetseiten und in Social-Media-Beiträgen des BIÖG, des Robert Koch-Instituts (RKI) und der Deutschen Krebsgesellschaft (DKG) sowie Hinweise der Krankenkassen. Weiterhin eignen sich lebensweltbezogene Maßnahmen, wie Schulprojekte, zur Impfkommunikation, um insbesondere auch schwer erreichbare Personengruppen zu adressieren. Eine Übersicht und Beispiele dazu liefert die Internetseite der Nationalen Lenkungsgruppe Impfen (NaLI; [33]).

Zudem sollten (medizinische) Fachakteurinnen und -akteure verstärkt eingebunden werden. Impfempfehlungen der STIKO werden fast ausschließlich durch niedergelassene Ärztinnen und Ärzte umgesetzt, wodurch ihnen eine bedeutsame Beratungsfunktion zukommt. Eine Stärkung der impfbezogenen Kommunikation durch gezielte Fortbildungs- und Informationsmaterialien kann Ärztinnen und Ärzte in ihrer Beratungsfunktion unterstützen.

Auch könnte ein Ausbau der Impfkommunikation innerhalb der Versorgungsstruktur für HPV-Impfungen die Impfquoten erhöhen. Ein wichtiger Ansatzpunkt ist die gesetzlich verankerte Jugenduntersuchung J1, die zwischen 12 und 14 Jahren erfolgt und die auch eine Beratung zur HPV-Impfung umfasst. Im Vergleich zu den etablierten U‑Untersuchungen, die Teilnahmequoten von über 90 % erreichen [34], beansprucht lediglich etwas weniger als die Hälfte der Jugendlichen die J1 [35, 36]. Eine Erhöhung der Teilnahme an der J1 könnte dazu beitragen, auch die HPV-Impfquoten zu steigern. Das BIÖG informiert Eltern und Jugendliche zur J1 und setzt umfangreiche Maßnahmen zur Steigerung der Bekanntheit der Untersuchung um [37]. Ein systematisches, flächendeckendes Einladungswesen, wie es bei den Früherkennungsuntersuchungen U1 bis U9 oftmals bereits existiert, könnte außerdem dazu beitragen, die Teilnahmequoten an der J1 zu erhöhen. Zudem wird die Einführung einer weiteren Früherkennungsuntersuchung (neue U10) vom Gemeinsamen Bundesausschuss (G-BA) geprüft [35]. Eine Erhöhung der Impfquoten könnte darüber hinaus durch systematische Impferinnerungssysteme gelingen [38, 39]. Die NaLI hat ein nationales Konzept mit strategischen Handlungsfeldern zur Förderung des Impfwissens und damit zur Steigerung der HPV-Impfquoten erarbeitet und stellt dieses auf ihrer Homepage zur Verfügung [33, 40].

### Limitationen

Die vorliegenden Ergebnisse sind unter Berücksichtigung von Limitationen zu interpretieren. Da es sich um eine Befragung von Personen aus einem Online-Access-Panel handelt, basiert die Stichprobe nicht auf einer Zufallsauswahl der Bevölkerung. Um dennoch eine Stichprobenstruktur zu erreichen, die in ihren Eigenschaften der Allgemeinbevölkerung ähnelt, erfolgte die Stichprobenziehung quotiert nach den Merkmalen Bildung, Alter, Geschlecht und Wohnort. Außerdem wurde die Stichprobe, angepasst an die Sollstrukturen des Mikrozensus, nach für Deutschland repräsentativen Kriterien gewichtet. Da zur Verteilung der sexuellen Orientierung keine Angaben im Mikrozensus vorliegen, wurde für dieses Merkmal nach der Verteilung innerhalb des Panels gewichtet. Als weitere Limitation sind Selektionseffekte zu nennen, die bei Online-Befragungen nicht auszuschließen sind. So können bestimmte Personengruppen, z. B. solche mit geringer Medienaffinität oder Lesekompetenz, unterrepräsentiert sein [32]. Zudem können Erinnerungslücken und sozial erwünschte Antworttendenzen die Ergebnisse verzerren. Letztere sind bei Online-Befragungen im Vergleich zu telefonischen Befragungen jedoch seltener zu erwarten [32]. Weiterhin ist zu beachten, dass es sich um Analysen von Querschnittsdaten handelt und somit keine Ableitung von Kausalzusammenhängen möglich ist.

## Fazit

Die Ergebnisse der LIEBESLEBEN-Studie verdeutlichen, dass in Deutschland trotz der geltenden Impfempfehlung für Mädchen und Jungen Wissensdefizite zur HPV-Impfung existieren. Es besteht Handlungsbedarf zum Aus- und Aufbau von Maßnahmen zur Impfkommunikation, um das Impfwissen zu fördern, die Impfquoten zu erhöhen und HPV-assoziierte Folgeerkrankungen zu verhindern. Solche Maßnahmen umfassen Informationsangebote für die Allgemeinbevölkerung und Zielgruppen, die (lebensweltbezogene) Ansprache der Zielgruppen, die Einbindung (medizinischer) Fachakteurinnen und -akteure sowie den Ausbau der Impfkommunikation innerhalb der Versorgungsstruktur. Unsere Ergebnisse legen außerdem nahe, dass verstärkt auf die präventiven Vorteile einer HPV-Impfung für Jungen fokussiert werden sollte. Männer sowie Personen mit geringer Bildung sollten besonders adressiert werden, da sie ein größeres Wissensdefizit aufweisen. Zur zielgruppenspezifischen Konzeption, Steuerung, Weiterentwicklung und Evaluation von Impfkommunikationsmaßnahmen sind regelmäßige Bevölkerungsbefragungen zur Bekanntheit der HPV-Impfung erforderlich.

## Data Availability

Der minimale Datensatz, der den Ergebnissen zugrunde liegt, ist im Forschungsdatenzentrum des Bundesinstituts für Öffentliche Gesundheit (BIÖG) archiviert und kann von Forschenden auf begründete Anfrage eingesehen werden. Anfragen können per E‑Mail an fdz@bioeg.de gestellt werden.
